# Vitamin D in pediatric age: Current evidence, recommendations, and misunderstandings

**DOI:** 10.3389/fmed.2023.1107855

**Published:** 2023-03-16

**Authors:** Antonio Corsello, Giulia C. I. Spolidoro, Gregorio P. Milani, Carlo Agostoni

**Affiliations:** ^1^Department of Clinical Sciences and Community Health, University of Milan, Milan, Italy; ^2^Pediatric Unit, Fondazione IRCCS Ca’ Granda Ospedale Maggiore Policlinico, Milan, Italy

**Keywords:** vitamin D, micronutrients, vitamin supplementation, pediatric screening, child public health, dietary supplements, infant nutrition, rickets

## Abstract

In recent years vitamin D has been in the spotlight of many researchers for its possible role in various disorders, including autoimmune and infectious diseases. Even if vitamin D deficiency remains a major public health problem, its symptomatic manifestations are less and less common in clinical practice, and pediatric age represents a “gray area” where vitamin D supplementation is often administered in the absence of an effective evaluation of its status. Moreover, a poor knowledge about different definitions of “deficiency,” “insufficiency,” and similar terms is spread among clinicians, while guidelines are not univocal, especially after the first year of life. The aim of this brief opinion paper is to sum up recent evidence about vitamin D status and its supplementation in pediatrics, in order to better clarify a common definition of its deficiency. The aim of this opinion article is to raise awareness on this topic among clinicians and encourage a discussion on the real need for routine 25-hydroxycholecalciferol serum evaluation and its supplementation.

## 1. Introduction

The role of vitamin D for calcium metabolism and, especially, for the treatment of rickets was first identified in 1922 by the American biochemist Elmer McCollum (1879–1967) (1). After his observations, thousands of papers have shed light on its important and multifaceted function for human health, not only regarding the musculoskeletal system (2, 3). At the same time, several investigators have observed that a surprising number of patients may present deficient levels of this micronutrient, regardless of their age and origin (4–6). Vitamin D deficiency remains a major public health problem, even after a century since its discovery (7).

At least two under-rated factors might contribute to the persistence of this issue: the lack of a common definition of vitamin D deficiency and the existence of partially contrasting recommendations on vitamin D prophylaxis.

The aim of this opinion article is to highlight recent evidence about vitamin D status and its supplementation in pediatrics, to better clarify a common definition of its deficiency. Our goal is to raise awareness among clinicians of this topic and encourage a discussion on the real need for routine 25-hydroxycholecalciferol serum evaluation and supplementation.

## 2. The effects in pediatric age: Bone health and immune function

Vitamin D plays an important role in bone growth and remodeling by osteoblasts and osteoclasts and is essential to maintain calcium, phosphate, and magnesium body homeostasis by regulating intestinal absorption, and renal absorption/excretion, alongside the parathyroid hormone (PTH). Plasma calcium and phosphate are mainly influenced by the active form of vitamin D (1α,25-dihydroxyvitamin D) and PTH, while a minor role is also attained by other humoral factors (8).

Magnesium is influenced, though to a lesser degree, by the same factors that control calcium, and it indirectly influences calcium by altering PTH synthesis and secretion in response to hypocalcemia and by assisting in the activation of vitamin D (9). Indeed, magnesium functions as a cofactor in many kidneys and liver enzymatic reactions and it is required by all the enzymes that intervene in vitamin D metabolization (10). Consequently, a lack of magnesium may result in disruption to the calcium/phosphate homestays regulated by active vitamin D/PTH and may cause vitamin D–resistant hypocalcemia (9, 10). On the other hand, vitamin D deficiency or overload may result in hypermagnesemia (magnesium not utilized) or hypomagnesemia (excessive consumption of magnesium).

In the early stages, vitamin D deficiency results in impaired calcium intestinal absorption and consequent low serum calcium levels (hypocalcemia). In turn, hypocalcemia stimulates PTH secretion which acts to normalize serum calcium by reducing renal calcium excretion, increasing renal phosphate excretion, and stimulating renal production of active vitamin D. High levels of PTH (hyperparathyroidism) also boost osteoclast activity which determines bone calcium release. The combination of low serum phosphate levels (hypophosphatasemia) and increased osteoclast activity results in bone demineralization (11).

In children, severe vitamin D deficiency may cause rickets, a childhood metabolic bone disease caused by under-mineralization of the growing bone. Following the growth plate closure, in older children and adults, the term “osteomalacia” is used to describe the demineralization of bone at sites of bone remodeling (12).

Vitamin D positive effects on the innate immune system are well known since the discovery of its historical beneficial effects on mycobacterium tuberculosis infection (13, 14). Vitamin D modulates monocytes, macrophages, dendritic cell responses, and the production of interleukins (15). Autoimmune diseases (ADs) are caused by an erroneous activation of the immune system, with subsequent destruction of tissues by autoreactive immune cells, which can react against self-antigens (16). Among the causes contributing to the development of ADs, an insufficient vitamin D serum concentration might play a significant role, as proven by epidemiologic findings of higher incidences of ADs among countries with lower sun exposures and high prevalence of vitamin D insufficiency (17, 18).

Recent studies found the vitamin D’s involvement in the suppression of T lymphocyte proliferation and adaptive immune system, causing a shift from a Th1 to a Th2 phenotype and a subsequent alteration in the differentiation and maturation of T cells, inducing T regulatory cells function and immune self-tolerance (19, 20). Moreover, B lymphocytes have been found to express vitamin D receptors, which, when activated, can inhibit the differentiation into plasma cells and modulate immunoglobulin production (21). All these effects could explain the possible connection between variable vitamin D serum levels and the probability to develop an AD (22).

Studies conducted among children with type 1 diabetes (T1D) found a significant difference in the incidence of disease according to the gradient of sun exposure in different countries and the seasonal variability (23, 24). Conflicting evidence has been found about the hypothesis that vitamin D supplementation could reduce the probability to develop T1D in early life, even if a protective correlation between minor incidence and higher serum levels has been found in some studies (25–27). Other studies observed a delay in the progression of beta-cells destruction when vitamin D is administered in an early stage of onset and diagnosis (28–30). Two meta-analyses found that the risk of developing T1D was significantly reduced in children supplemented with vitamin D (27, 31). For these reasons, avoiding a vitamin D insufficiency in patients at high risk for developing T1D is suggested, even if evidence for a specific supplementation in these subjects is lacking (17). Concerning other ADs, low 25(OH)D sera levels have been associated with the development of autoimmune thyroiditis, such as Hashimoto’s disease and Grave’s disease, both in children and adults (32–34).

Vitamin D also acts as an enhancement of the intestinal defense mechanisms, locally regulating the mucosal immune system and preventing harmful microbial proliferation (35, 36). For instance, an association between lower vitamin D concentrations and an increased risk of developing IBD has been suggested (37–40). This evidence can be strengthened by the reduced vitamin receptors expression found in the colon mucosa of mice affected by IBD when compared to healthy controls, with an increased production of Th1 and Th17 lymphocytes and inflammatory cytokines in the gastrointestinal tract (41, 42). For these reasons, according to recent interventional studies, a vitamin D supplementation can be considered a potential effective and safe therapeutic choice in patients with IBD (43–46).

Emerging evidence from various meta-analyses supposes a plausible role of low vitamin D levels in many other ADs, such as rheumatoid arthritis, systemic lupus erythematosus, and multiple sclerosis, and vitamin D receptors polymorphisms have been associated with higher incidences of several ADs (47–51). However, optimal vitamin D concentrations that could reduce the possible occurrence of ADs are not clear yet, even if levels higher than 20 ng/mL (about 50 nmol/L) should be considered sufficient to maintain the physiologic calcemic and non-calcemic functions of vitamin D (52).

## 3. Deficiency and insufficiency: Words not to be confused

Vitamin D deficiency has been recognized in a wide range of pathological conditions, from allergic to immune-mediated diseases such as diabetes or chronic inflammatory bowel diseases (25, 27, 53–55). More recently, vitamin D deficiency and its eventual supplementation have been even considered linked with COVID-19 symptoms and prevention, generally without any proof of a cause-and-effect relationship, including reverse causality (56–59).

Although cases of rickets and clinical manifestations of symptomatic vitamin D deficiency are actually rare, many studies worldwide have shown high rates of vitamin D deficiency and insufficiency (measured as the serum concentration of 25-hydroxycholecalciferol, 25(OH)D) in the pediatric population, with rates ranging on average between 40 and 75%, even in developed countries (60–63). The low rates of sunlight exposure in Nordic countries and the limited production in Southern ones, related to the natural protection of dark skin phenotypes toward light together with a diffused poor nutritional status, may represent possible cofactors of this evidence (64–66). Variable percentages of insufficiency/deficiency reported in the literature may be linked also to the variability of the screening methods, which often use improper terminologies and cut-offs. The universal application of unnecessary (and expensive) routine supplementations in a healthy population does not have a scientific rationale. Moreover, there is currently no scientific evidence to recommend vitamin D supplementation in the treatment and prevention of diseases like asthma, allergic, or immune disorders (67).

Consequently, it might be considered if and how a vitamin D deficiency may be responsible for so many different diseases. A meaningful point is represented by the lack of knowledge among clinicians about the current reference values of hypovitaminosis, probably related to the frequent and ambiguous use of terms such as “deficiency,” “sufficiency,” “adequacy,” or “insufficiency” (68, 69). As a consequence, surveys conducted on the general population and based on inappropriate cut-off values have sometimes led to unnecessary supplementations in healthy populations (52, 70, 71).

The threshold value about hypovitaminosis (and therefore “deficiency,” and not “insufficiency”) is debated (72–74), but according to some authorities we suggest considering adequate levels above 30 ng/mL (75 nmol/L). Values included in the range of 20–29 ng/mL (50–74 nmol/L) are then to be considered “insufficient” (Table 1) (75, 76). Furthermore, with the exception of subjects at risk of hypovitaminosis or those who may require supplementation (e.g., inadequate dietary intake), no screening of serum 25(OH)D dosages is recommended, nor an arbitrary or medically unjustified exogenous administration should be generally performed before verifying an effective deficiency/insufficiency (77, 78).

**Table 1:** Suggested cut-off values for the definition of vitamin D status based on circulating levels of 25(OH)D (74).

	Severe deficiency	Deficiency	Insufficiency	Sufficiency
Serum	<10 ng/mL	<20 ng/mL	20–29 ng/mL	≥ 30 ng/mL
25(OH)D	(<25 nmol/L)	(<50 nmol/L)	(50–74 nmol/L)	(≥ 75 nmol/L)

## 4. Risk factors and current evidence

Several risk factors are associated with vitamin D deficiency in children and may be considered by clinicians upon deciding whether blood serum screening and/or vitamin D supplementation should be started.

The levels of vitamin D may also be influenced by ethnicity, genetic predispositions, and skin types, with darker skin types requiring more sunlight exposure to absorb vitamin D (72, 79–81). Lastly, there is a risk for vitamin D deficiency also for pediatric and adult subjects who are treated with certain medications (e.g., anti-epileptics, and glucocorticoids) and for patients with chronic diseases which prevent vitamin D intestinal absorption, such as celiac disease or cystic fibrosis, but also diseases which impact the liver, or the kidneys, preventing physiologic vitamin D metabolism (72, 82–85).

Another major risk factor of vitamin D deficiency is the insufficient sunlight exposure, which affects the body’s capacity to produce vitamin D (86, 87). Therefore, spending most of the time indoors or living in regions where the weather is mostly cloudy, and cold represents a risk for vitamin D deficiency. Accordingly, other factors such as latitude, and altitude, which directly impact the sun angle and ultraviolet radiations may impact vitamin D status (72).

Another known risk factor for vitamin D deficiency is related to age, with infants being more at risk of developing hypovitaminosis. Under 12 months of age, the risk is even higher for premature babies, who have less vitamin D deposits, and in children who are breastfed (88). Indeed, human breast milk is almost lacking in proper amounts of vitamin D, with concentrations that range from 10 to 80 IU/L in healthy lactating women, depending on the method of measurement (88, 89).

Considering the first 6 months of age, mothers who are lacking in vitamin D, supply even fewer amounts to their infants, while, higher breast milk quantities of vitamin D positively correlate with higher mother 25(OH)D serum levels (90–92). The rapid conversion of cholecalciferol to 25(OH)D in the mother’s liver represents another obstacle to a high passage of vitamin D, and it is maybe one of the main reasons for the 0.2 ratio between maternal and breast milk 25(OH)D (93).

The main problem of infants exclusively breastfed by mothers supplemented with 400 IU/day, the typical amount assumed during pregnancy, is the typical deficiency reported in this population (94, 95). Moreover, breastfed infants typically lack in adequate sunlight exposure and the administration could be affected by poor parental compliance (96, 97).

When exclusive breastfeeding is no longer sufficient to meet all the nutritional needs, it is, therefore, necessary to introduce other foods and liquids. This period is known as “complementary feeding” or “weaning,” and should generally be started after 6 months of life, with partial breastfeeding that may continue approximately up to 2 years of age (98).

Low dietary intake of vitamin D remains a common reason for vitamin D deficiency even in older children/adolescents, especially in overweight and obese individuals. Moreover, overweight and obese subjects are more at risk of low blood levels of vitamin D, as adiposity may prevent vitamin D release from the deposits in the fatty tissue and therefore its bioavailability (66, 99).

## 5. International recommendations

For all the mentioned reasons, most international and national guidelines actually recommend vitamin D prophylaxis to all infants during the first year of life, in order to prevent possible deficiency conditions and in consideration of the frequent, unpredictable, and often insufficient supply typical of the early infancy (73, 74). It has been estimated that infant formulas are able to provide appropriate amounts of vitamin D (400 IU/day) only once an average intake of at least 1 l of milk per day has been reached, which generally takes place when the baby’s body weight is more than 6 kg (100). Moreover, even if cholecalciferol (vitamin D_3_) seems to be more effective in raising 25(OH)D levels, there is no proven difference between the administration of ergocalciferol (D_2_) or D_3_ formulation in preventing possible deficiencies (101, 102). Dosages above 400 IU/day did not show significant differences in vitamin D sufficiency rates and bone health during the first year of life (94). Higher intakes have been suggested only in preterm births; in fact, the European Society of Pediatric Gastroenterology, Nutrition, and Hepatology (ESPGHAN) recommends prophylaxis with 800–1,000 IU/day (avoiding a pro kg dosage) during the first months of the preterm, in order to quickly correct the reduced infants’ levels of 25(OH)D, thus reducing the risk of symptomatic deficiencies (103). As for breastfeeding, although some recommendations advise for maternal supplementation of 600 IU per day, some data suggest that this practice does not significantly change the concentration of 25(OH)D in breast milk, which is generally poor in vitamin D (93). For this reason, the American Academy of Pediatrics recommends supplementing all breastfed and partially breastfed infants, regardless of maternal supplementation (88). However, adherence to these recommendations is low (104). Furthermore, due to the poor evidence to assess the benefits of supplementation for improving maternal and infant health outcomes, the World Health Organization (WHO) does not recommend routine vitamin D supplementation in pregnant women (105).

In consideration of the low levels of adherence to a daily administration of vitamin D found in many populations that differed for geographic and socioeconomic conditions, alternative approaches to a daily supplementation based on high doses (up to 6,400 IU/day) given directly to fully breastfeeding mothers have been found to be safe and equally effective in maintaining adequate levels of 25(OH)D, as well as monthly administrations of 50,000 IU given directly to infants (94, 104, 106–110).

After 12 months of life, evidence about pediatric vitamin D supplementation needs is less clear and univocal (111–114). A case-by-case evaluation of the toddler, child, and young adult lifestyle has then been recommended, to correct possible modifiable risk factors when present, particularly in adolescents, by virtue of their increased needs related to major skeletal growth and also the pubertal spurt (99). Any supplementation is therefore only indicated in cases of poor sun exposure or in specific conditions (111). This approach is also supported by WHO, which focuses the indication on vitamin D supplementation more on geographical location and sun exposure than on child age (115). As for the administration of fortified foods in early childhood, the consumption of vitamin D-fortified milk between 2 and 6 years may represent a safe and significantly effective nutritional measure in preventing 25(OH)D insufficiency during periods of reduced sunlight exposure (116–119). On the other hand, other societies, such as the Endocrine Society and the Institute of Medicine, recommend an average vitamin D intake of 600–1,000 IU/d between 1 and 18 years of age (120, 121).

Considering these discrepant recommendations, the inconclusive data on this issue, and that universal screening of 25(OH)D serum levels cannot represent a feasible solution, new studies should assess if vitamin D evaluation in selected cases would be more appropriate and cost-effective than a routinary, continued, and widespread supplementation.

As was observed 10 years ago in the Consensus statement by the ESPGHAN Nutritional Committee, data on vitamin D concentration and vitamin D deficiency in healthy pediatric subjects are limited, with a lack of consistency in terms of study design and definition of vitamin D deficiency (72). From the studies examined in the statement, it appears that a considerable number of healthy European children and adolescents may be expected to be vitamin D deficient (122–134).

Besides, routine vitamin D screening in healthy children is currently not recommended by experts globally, which partially explains the lack of data and systematization of literature regarding vitamin D status in healthy subjects (73).

Finally, although there are few reports on possible damages from high levels of exogenous administration of vitamin D for example, in cases of mutation of the 24-hydroxylase gene or in sporadic cases of overdosages, possible conditions such as hypercalcemia and hypercalciuria might occur, leading up to acute symptoms such as pain, fever, chills, anorexia, and later to soft tissue calcification, and related chronic pathologies such as nephrocalcinosis in case of prolonged overdosages (135, 136). The issue is still discussed in the hypothesis of either a rare genetic predisposition, or real differences in comparison with the expected spontaneous epidemiological distribution of cases. Cases of vitamin D intoxication have been described only for serum levels above 150 ng/mL (137).

It should be once more stressed that vitamin D levels can significantly vary depending on the lifestyle and overall exposure to ultraviolet B rays, which is affected by factors such as geographic location, age, skin, and family habits (66, 138, 139). In newborns and infants, other factors may well affect vitamin D status such as season of birth, maternal prophylaxis in pregnancy, type of breastfeeding, human milk variable individual composition, and the family socioeconomic status (140, 141). Recently, the rising rate of obesity and the lipophilic nature of vitamin D have suggested the need for higher quantities of vitamin D for these subjects, up to at least 1,000–1,500 IU/day (99, 142).

The 25(OH)D serum dosage should be cautiously considered in clinical practice. The National Institute for Health and Care Excellence (NICE) guidelines recommend dosing 25(OH)D serum only in patients with signs of hypovitaminosis, or in the presence of documented risk factors that justifies their dosage (143). Instead, a 6-month 25(OH)D dosage may be appropriate in supplemented patients to evaluate its effectiveness or to consider whether to suspend it.

In Table 2, we summarize different guidelines and statements for supplementation.

**Table 2:** Different guidelines for vitamin D supplementation.

First author, year	Title	Type	Vitamin D status cut-off values considered	Vitamin D toxicity level considered	Population	Vitamin D supplementation
Munns et al., 2016 (73)	Global Consensus Recommendations on Prevention and Management of Nutritional Rickets	Consensus statement from expert member of various international societies	Sufficiency, >50 nmol/L Insufficiency, 30–50 nmol/L Deficiency, <30 nmol/L	>250 nmol/L, with hypercalcemia, hypercalciuria, and suppressed PTH	Children and adults	Infants (0–12 months) 400 IU/day in the first year of life, independent of their mode of feeding. Subject beyond 12 months of age: 600 up to 2000 IU/day (minimum duration 12 weeks) in children with history of symptomatic vitamin D deficiency requiring treatment or children and adults at high risk of vitamin D deficiency Pregnant women 600 IU/d throughout pregnancy
Pludowski et al., 2022 (76)	Clinical Practice in the Prevention, Diagnosis, and Treatment of Vitamin D Deficiency: A Central and Eastern European Expert Consensus Statement	Consensus statement by Eastern European expert	Sufficiency 30–50 ng/mL (75–125 nmol/L) Insufficiency between ≥20 ng/mL (≥50 nmol/L) and < 30 ng/mL (<75 nmol/L) Deficiency <20 ng/mL (<50 nmol/L)	>100 ng/mL (250 nmol/L)	Adults	Healthy adults 800 up to 2000 IU/day in: (1) healthy subjects who want to achieve a targeted/measured 25(OH)D concentration, during wintertime (2) subjects >65 years throughout the year (3) Pregnant women or planning a pregnancy Subjects with medical conditions up to 4,000 IU/day
Braegger et al., 2013 (72)	Vitamin D in the healthy European pediatric population	Consensus statement from member of the ESPHGAN Committee of nutrition	Sufficiency >50 nmol/L Severe deficiency <25 nmol/L.	“No agreement on a vitamin D toxicity threshold”	Children	Infants (birth to 12 months) 400 IU/day in the first year of life Beyond 12 months: >600 IU/day supplementation in children from identified risk groups
Saggese et al. 2018 (99)	Vitamin D in pediatric age: consensus of the Italian Pediatric Society and the Italian Society of Preventive and Social Pediatrics, jointly with the Italian Federation of Pediatricians	Consensus statement form national Pediatric societies in Italy	Sufficiency ≥30 ng/mL (≥ 75 nmol/L) Insufficiency 20–29 ng/mL (50–74 nmol/L) Deficiency <20 ng/mL (<50 nmol/L) Severe deficiency <10 ng/mL (<25 nmol/L)	Not reported	Children	Preterm infants: (1) 400–800 IU/day for preterm infants with weight ≥ 1,500 g (2) 200–400 IU/day by enteral feeding in preterm infant with weight < 1,500 g Infants: 400 IU/day in all newborns independent of the type of feeding. Subjects beyond 12 months of age: 600 up to 1,000 IU/day in children and adolescents with risk factors for vitamin D deficiency
Agostoni et al. 2010 (103)	Enteral Nutrient Supply for Preterm Infants: Commentary from the European Society of Paediatric Gastroenterology, Hepatology, and Nutrition Committee on Nutrition	Commentary from ESPHGAN Committee of nutrition	N/A	Not reported	Children	Preterm infants 800 up to 1,000 IU/day during the first months of life
Wagner et al., 2008 (88)	Prevention of Rickets and Vitamin D Deficiency in Infants, Children, and Adolescents	Guidelines from the American Academy of Pediatrics	“Serum 25-OH-D concentrations in infants and children should be ≥50 nmol/L (20 ng/mL).”	Not reported	Children	Infants 400 IU/day in the first year of life in breastfed or partially breastfed infants, and in non-breastfed infants who are ingesting <1,000 mL/day of vitamin D–fortified formula or milk Older children 400 IU/day in case of diet with <1,000 mL/day of vitamin D–fortified formula or milk Adolescents 400 IU/day in case the individual does not obtain 400 IU of vitamin D per day through vitamin D–fortified milk/vitamin D–fortified foods (e.g., cereals) Children/adolescents with risk factors: 400 IU/day supplement despite a food intake of 400 IU/day
Rogers et al., 2012 (105)	Vitamin D supplementation in pregnant women	WHO guidelines	N/A	Not reported	Pregnant women	Supplementation during pregnancy as part of routine antenatal care not recommended (conditional recommendation) In cases of documented deficiency, vitamin D supplements may be given
Holick et al., 2011 (121)	Evaluation, Treatment, and Prevention of Vitamin D Deficiency: an Endocrine Society Clinical Practice Guideline	Clinical practice guidelines by the Endocrine Society	Insufficiency 21–29 ng/mL (525–725 nmol/L) Deficiency <20 ng/mL (50 nmol/L),	Not reported	Children and adults	Infants aged 0–1 year who are vitamin D deficient 2000 IU/day for 6 weeks followed by maintenance therapy of 400–1,000 IU/day Children aged 1–18 years who are vitamin D deficient 2000 IU/day for at least 6 weeks followed by maintenance therapy of 600–1,000 IU/day Adults who are vitamin D deficient 6,000 IU/day for 8 weeks followed by maintenance therapy of 1,500–2000 IU/day

## 6. Conclusion

All children during the first year of life should receive an oral supplementation of vitamin D. Beyond this age, geographic and cultural variability should always be considered by clinicians and scientific societies in recommending, or not, the need for supplementation. Vitamin D status may be closely connected to social inequities and related diseases. Moreover, constant and unjustified monitoring of vitamin D levels has then been considered unnecessary in the general population. Screening of serum 25(OH)D concentration should be carried out only in selected cases.

## Author contributions

All authors listed have made a substantial, direct, and intellectual contribution to the work and approved it for publication.

## Funding

This study was partially funded by Italian Ministry of Health—Current research IRCCS.

## Conflict of interest

The authors declare that the research was conducted in the absence of any commercial or financial relationships that could be construed as a potential conflict of interest.

## Publisher’s note

All claims expressed in this article are solely those of the authors and do not necessarily represent those of their affiliated organizations, or those of the publisher, the editors and the reviewers. Any product that may be evaluated in this article, or claim that may be made by its manufacturer, is not guaranteed or endorsed by the publisher.
